# Correction to “In Vivo Optical Assessment of Cerebral and Skeletal Muscle Microvascular Response to Phenylephrine”

**DOI:** 10.1096/fba.2025-00315

**Published:** 2025-12-22

Mawdsley L, Eskandari R, Kamar F, et al. “In Vivo Optical Assessment of Cerebral and Skeletal Muscle Microvascular Response to Phenylephrine,” *FASEB BioAdvances* (2024); 6: 390–399, https://doi.org/10.1096/fba.2024‐00063.

In paragraph 2 of Section 2.1: Animal Protocol, the reported dosage for the phenylephrine injections was incorrect. The correct intravenous dosage is 10 μg/mL.

We apologize for this error.

